# Factors associated with persistent hypertension after puerperium among women with pre-eclampsia/eclampsia in Mulago hospital, Uganda

**DOI:** 10.1186/1471-2393-10-12

**Published:** 2010-03-12

**Authors:** Emmanuel B Ndayambagye, Miriam Nakalembe, Dan K Kaye

**Affiliations:** 1 Department of Obstetrics and Gynecology, Makerere University Medical School, PO Box 7072, Kampala, Uganda

## Abstract

**Background:**

Women with severe pre-eclampsia/eclampsia are at risk of developing chronic hypertension in future. Chronic hypertension may manifest initially as persistent hypertension at the end of the puerperium. The objective was to determine the incidence and maternal biochemical, hematological and socio-demographic risk factors for persistent hypertension in patients with pre-eclampsia/eclampsia.

**Methods:**

This was a prospective cohort study conducted from November 2008 to May 2009 at Mulago hospital labor ward and postnatal clinic. Participants were 200 women managed for pre-eclampsia/eclampsia and followed up to the end of the puerperium. Data was collected through using pre-coded interviewer-administered questionnaires, checking medical records and laboratory investigations. STATA (release 9) software was used for data analysis. At bivariate analysis, the relative risk of persistent hypertension was estimated at the 95% confidence level. Using multivariate logistic regression analysis, factors that were independently associated with persistent hypertension were evaluated.

**Results:**

Fifty four (27.7%) out of the total 195 women had persistent hypertension after puerperium. Serum creatinine and the age of the patient were the only factors associated with persistence of hypertension after puerperium.

**Conclusion:**

Nearly every one in four mothers with pre-eclampsia/eclampsia are at risk of persistent hypertension after the puerperium. Serum creatinine, serum uric acid and participants' age were the only factors independently associated with persistence of hypertension after the puerperium.

## Background

Pre-eclampsia is an idiopathic multi-system disorder specific to pregnancy. It is characterized by hypertension (a blood pressure of higher of 140/90 mmHg or higher in a previously normotensive women) and proteinuria. Pre-eclampsia and eclampsia complicate about 6-8% all pregnancies worldwide [1] and are associated with increased maternal morbidity and mortality. They are also associated with development of chronic hypertension [2] as well as cardiovascular and renal complications later in life. Women with pre-eclampsia/eclampsia are at higher risk of obstetric complications like abruptio placenta and intra-uterine growth restriction [2] as compared to normotensive women. They also have a higher risk of developing chronic hypertension in the future (14.8% versus. 5.6%), and this risk increases markedly on long-term follow up (to 51% versus 14%) [2].

Because they are at risk of developing chronic hypertension, all women with pre-eclampsia/eclampsia require follow up after delivery. Factors that predict chronic hypertension include maternal age and gestation age of onset of pre-eclampsia [3]. A higher maternal age [4] and lower gestation age at onset increase risk of chronic hypertension. During the postnatal period, the hypertension and proteinuria due to pre-eclampsia/eclampsia resolve within six weeks, and women having persistent hypertension and proteinuria thereafter may have an underlying cause. Postnatal review at 6-12 weeks after delivery provides an opportunity to ensure that manifestations of pre-eclampsia and any associated systemic complications have resolved. Early identification of chronic hypertension among these women during follow-up might prevent or reduce its long-term complications if relevant interventions to decrease or avert associated renal or cardiovascular damage are instituted in time. In Mulago hospital, about 3-4 patients with pre-eclampsia are seen per day, constituting 8.2% of all labour ward admissions. The objective of the study was to evaluate the incidence and factors associated with persistent hypertension in patients that present with pre-eclampsia/eclampsia.

## Methods

Being Uganda's national referral hospital, Mulago receives women booked with the hospital's antenatal clinic, self referrals and referrals from other health units. The hospital conducts about 50-65 deliveries daily, over 25,000 deliveries per year. This was a prospective cohort study carried out in new Mulago hospital's labor ward and postnatal clinic, from 18^th ^November 2008 to 17^th ^March 2009. Participants were women admitted in New Mulago hospital with pre-eclampsia or eclampsia. These were followed up to the end of the puerperium. Hypertension in pregnancy was defined as any of the following: i) a blood pressure measurement of higher than or equal to 140 mmHg systolic or 90 mmHg diastolic (measured at rest on at least 2 occasions in a patient of more than 20 weeks of gestation); ii) a rise in systolic pressure of 30 mmHg or higher and diastolic pressure of 15 mmHg or higher, ((from the pre-pregnancy or first trimester measurement, measured on at least 2 occasions 4 hours apart,). All women with essential hypertension, chronic hypertension, hypertension in a prior pregnancy, a history of hypertension in the first trimester history, renal disease or pre- eclampsia in a prior pregnancy were excluded from the analysis.

Pre-eclampsia was defined as presence of hypertension (as defined above) and albuminuria (on urinary dipstick examination). A diagnosis of mild pre-eclampsia was made when a blood pressure of less than 160/110 mmHg was associated with proteinuria (albuminuria) of 2+ or less on urinary dipstick examination, at any time during the management and subsequent follow up. Severe pre-eclampsia was diagnosed as a blood pressure of 160/110 mmHg or higher in association with proteinuria of 3+ or more on urinary dipstick examination, at any time during the management and subsequent follow up. A diagnosis of eclampsia was made when the patient with features of pre-eclampsia developed convulsions (tonic-clonic seizures). A diagnosis of HELLP syndrome was made where the patient presented with hypertension, elevated hepatic lactate dehydrogenase enzymes (greater than 600 units/liter), elevated aspartate and alanine transaminase enzymes (above 50 international units per liter) and platelet count less than 150,000 cells per mm3. (All patients with HELLP syndrome had features of severe pre-eclampsia. Presence of microangiopathic hemolytic anemia was not evaluated). The diagnosis of persistent hypertension was made when hypertension (as defined above) was found at any time six weeks or later postpartum.

### Data collection

Data was collected through interviews using an interviewer-administered questionnaire. Other data was collected by participant examination, biomedical investigations and review of participants' medical records. This data included social-demographic factors (age, parity, marital status, spouse's age,); obstetric factors (such as gestational age at presentation, gestation age at disease onset, family history of hypertension or pre-eclampsia and severity of pre-eclampsia. Biochemical data included serial uric acid levels, creatinine levels, platelet count, urinary protein levels and results of renal function tests). Blood pressure was measured using the manual sphygmomanometer machine in a sitting position. Urine protein was determined by dipstick examination. The primary outcome was persistence of hypertension at six weeks postpartum. The sample size of 195 was estimated using the Kish and Leslie formula (1965) where N = Z^2^P(1-P)/D,^2 ^and assuming that the incidence of chronic hypertension on a follow up of women with pre-eclampsia/eclampsia is about 14.8% [2].

All participants received antihypertensive and magnesium sulphate therapy according to a standard protocol as follows,: Magnesium sulphate 10 g intramuscularly together with 4 g intravenously were used as loading dose and 5 g intramuscularly every 4 hours until 24 hours later or as maintenance dose for all patients with severe pre-eclampsia and eclampsia. All patients with diastolic pressure above 100 mmHg received hydrallazine injection 5 mg every 30 minutes until the diastolic was less than 100 mmHg. Antihypertensive treatment was thereafter maintained with sublingual Nifedipine 20 mg. Continuous bladder drainage was instituted throughout the patients' hospital stay. The subsequent care and mode of delivery followed standard obstetric practice for pre-eclampsia/eclampsia.

### Data analysis

At analysis, characteristics of participants with persistent hypertension and those who were normotensive at six weeks postpartum were compared. Baseline characteristics of the participants were compared using *Pearson's *chi-square test for categorical data and Student *t*-test for numerical data. To adjust for confounding and interaction, multivariate logistic-regression analysis was conducted to determine factors that were independently associated with the development of persistent hypertension, using STATA software. During modeling for regression analyses, all variables of clinical importance or with p-value 0.2 and less on bivariate analysis were considered for inclusion. Persistent hypertension entered as *present = 1, absent = 0*. Parity, number of living children and age of spouse were entered as numerical variables. Participant age was evaluated as a numerical variable and as 5-year age categories. The model goodness-of-fit of the final logistic regression models was assessed by *Pearson's *chi-square test.

### Ethical Considerations

The study was approved by the research and ethics committees of Makerere University Faculty of Medicine and Mulago hospital. The participants gave written informed consent to participate in the study, whereby none were to be denied any healthcare in case they declined to participate or withdrew from the study. Medication and care for the pre-eclampsia/eclampsia were provided free to all participants.

## Results

During the study period, 200 participants with pre-eclampsia/eclampsia were recruited into the study. We analyzed data for 195 participants: two participants died during follow up, while three failed to turn up for postpartum review in the postnatal clinic. Of the participants, 25 (12.8%) had mild pre-eclampsia, 160 (82.1%) had severe pre-eclampsia and 10 (5.1%) had eclampsia. Only 150 patients (76.9%) had full evaluation for HELLP syndrome, and of these, only 6 (3.1%) had HELLP syndrome. Out of 195 participants, 54 (27.7%) had persistent hypertension and 141(72.3%) were found to have normal blood pressure at six weeks postpartum.

Table 1 shows the socio-demographic characteristics of the participants. Except for participants' age, there were no statistically significant differences in baseline characteristics of participants (between those who had persistent hypertension and those who were normotensive at six weeks postpartum) (p-value > 0.05). Likewise, the difference in family history of hypertension, family history of diabetes, history of pre-eclampsia among siblings and lifestyle factors (smoking and alcohol drinking) was not statistically significant.

**Table 1:** Comparison of baseline characteristics of participants with and without persistent hypertension

Characteristics	Normal Blood Pressure at 6 weeks postpartum		Persistent hypertension 6 weeks postpartum		P-value
	n = 141		n = 54		
	n	(%)	n	(%)	
*Age in complete years*					
15-19	36	(25.5)	3	(5.6)	
20-24	49	(34.6)	18	(33.3)	0.001
25-29	35	(24.8)	12	(22.2)	
30-34	16	(11.3)	13	(24.1)	
35-39	3	(2.1)	7	(12.6)	
40 years and above	2	(1.4)	1	(1.9)	
*Education*					
No formal education	4	(2.9)	1	(1.2)	
Primary	60	(42.9)	21	(39.6)	
Secondary	58	(41.4)	29	(54.7)	0.187
Tertiary	18	(12.9)	2	(3.8)	
*Occupation*					
Peasant/house wife	92	(65.2)	35	(64.8)	
Business woman	35	(24.8)	15	(27.8)	0.325
Unemployed	14	(9.9)	4	(7.4)	
					
*Marital status*					
Single	30	(21.3)	8	(14.8)	
Married	111	(78.7)	46	(85.2)	0.640
*Family history of Hypertension*					
Yes	65	(46.1)	32	(59.3)	
No	76	53.9)	22	(40.7)	0.230
*History of Diabetes*					
Yes	20	(14.2)	11	(20.4)	
No	121	(85.8	43	(79.6)	0.570
*History of pre-eclampsia among sisters*					
Yes	26	(17.3)	10	(19.2)	0.752
No	115	(82.7)	42	(80.8)	
*History of smoking*					
Yes	4	(2.8)	2	(1.9)	0.714
No	137	(97.2)	52	(98.1)	
*History of alcohol*					
Yes	42	(29.8)	18	(33.3)	0.574
No	99	(72.2)	36	(66.6)	

Table 2 compares the serum creatinine levels, seum uric acid levels, platelet count and urine protein levels at admission and after the puerperium among participants who had persistent hypertension and those who had become normotensive. *Serum uric acid*: the mean values of serum uric acid at admission were 417.7 (mg per 100 mls) versus 365.1 as compared to 317.6 versus 275.3 after six weeks postpartum. Although, at admission, the uric acid levels were higher among those who developed persistent hypertension, the difference between the two groups was not statistically significant. The difference in mean values of serum uric acid for those normotensive and those with persistent hypertension was statistically significant at 6 weeks postpartum.

**Table 2:** Comparison of laboratory results (mean values) at admission and after 6 weeks for the two groups

Variables Periods	Normal BP Mean plus standard deviation	Abnormal BP (Persistent Hypertension) Mean plus standard deviation	P-value
*† Serum uric acid (μmol/l)*	365.1 ± 330.1	417.7 ± 200	0.27
At admission	275.3 ± 200.6	317.6 ± 101.9	0.002
After 6 weeks			
*† Mean Platelet level (×10^9^/liter)*	202.4 ± 79.6	228.9 ± 96	0.051
At admission	241.9 ± 59.2	268.1 ± 79	0.014
After 6 weeks			
*† Serum Creatinine (mg/100 ml)*		948 ± 67.5	0.004
At admission	72.6 ± 38.4	251.7 ± 1289	0.071
After 6 weeks	55.8 ± 21.3		
*† Serum Urea (mg/100 ml)*		10.7 ± 24.3	0.066
At admission	5.5 ± 14.3	8.6 ± 17.9	0.361
After 6 weeks	6.5.0 ± 12.5		
*Urine protein at admission			
+	1.0 (0.7)	0.0 (0.0)	0.165
++	60 (42.6)	22 (40.7)	
+++	57 (40.4)	16 (29.6)	
++++	23 (16.3)	16 (29.6)	
*Urine protein after 6 weeks postpartum			
Negative	104 (74.3)	26 (48.2)	0.000
Trace	22 (15.7)	4 (7.4)	
+	8 (5.7)	16 (29.6)	
++	2 (1.4)	3 (3.6)	
+++	4 (2.9)	5 (9.3)	

Key: *Figures indicate numbers and percentages of participants in both groups

†Figures indicate mean values and the standard deviation

Units for serum creatinine and urea are mg per 100 mls, while those for platelet count are (× 10^9 ^per liter.

*Platelet count*: for the platelet count, the mean values at both admission and six weeks postpartum were significantly higher in the group that had persistent hypertension.

*Serum urea and creatinine*: Though overall, the mean serum urea and creatinine levels were higher at admission and end of the puerperium in the group with persistent hypertension (as compared to the group who became normotensive), only the difference in mean levels at admission was statistically significant. *Urine protein levels: *Though urine protein levels at admission were comparable for the two groups (those who became normotensive and those with persistent hypertension) at recruitment, they were higher among those with persistent hypertension. For example, there was 9.3% with proteinuria of 3+ among those with persistent hypertension compared to 2.9% among those normotensive after six weeks postpartum. Overall 44.4% of those with persistent hypertension had some degree of proteinuria, implying a strong association between postpartum proteinuria and persistent hypertension six weeks postpartum.

Table 3 shows the independent factors associated with persistence of hypertension. Participant age (especially among those in the category of 30-34 years) was statistically significant in predicting persistent hypertension. Serum creatinine at admission and urine protein at six weeks postpartum were significantly associated with persistent hypertension.

**Table 3:** Predictors of persistent hypertension after 6 weeks

Predictors	Risk ratio	Confidence interval (CL)	P-value
Age	2.61	1.31 - 8.40	0.027
15-19	2.43	0.75 - 7.87	0.138
20 -24	4.16	1.30 - 13.33	0.017
25 - 29	7.23	2.29 - 22.85	0.001
30 - 34	2.99	0.41 - 21.77	0.278
35 - 39	1.49	0.84 - 2.63	0.172
40-44			
Smoking	1.79	0.48 - 6.67	0.382
Family history of hypertension	0.87	0.55 - 1.38	0.555
History of alcohol	0.92	0.58 - 1.47	0.701
History of pre-eclampsia	1.06	0.67 - 1.67	0.717
Serum creatinine at admission	2.04	1.35 - 3.09	0.001
Serum urea	0.90	0.57 -1.44	0.672
Urine protein at admission	1.64	0.99 -2.70	0.051
Serum uric acid at end of puerperium	2.70	1.30 - 6.67	0.035

## Discussion

The study assessed the incidence of persistent hypertension and factors associated with persistent hypertension in patients with pre-eclampsia/eclampsia. The study shows that the incidence of persistent hypertension is 27.7% (54/195). Participants' age (especially among women aged 30-34 years), serum creatinine at admission, serum uric acid six weeks postpartum and urine protein at six weeks postpartum were the factors independently associated with persistent hypertension. Family history of hypertension, history of diabetes and history of pre-eclampsia among siblings were not significantly associated with persistent hypertension.

The finding of higher mean serum uric acid levels and higher serum creatinine levels as well as urine protein in the women with persistent hypertension might result from renal dysfunction in pre-eclampsia. During normal pregnancy, the increased blood volume leads to marked reduction in serum uric acid and creatinine levels. Renal blood flow and glomerular filtration rate are also markedly increased. In pre-eclampsia, renal perfusion and glomerular filtration are markedly reduced secondary to renal dysfunction. While the definite cause of renal dysfunction in pre-eclampsia remains unclear, one possible mechanism might be damage caused by endothelin, a potent endogenous vasoconstrictor peptide produced by endothelial cells [5]. Consequently, plasma uric acid levels are elevated in proportion to severity of pre-eclampsia. The renal insult secondary to vasospasm might be one of several potential factors involved in the pathogenesis of chronic hypertension among these women with pre-eclampsia. Likewise, chronic hypertension and pre-eclampsia may have similar or related risk factors. In women in whom endothelin levels are persistently elevated, serum uric acid is increased and creatinine clearance is diminished [5]. Elevated serum uric acid levels, which are prognostic for severe pre-eclampsia and eclampsia, are indicators of disease severity and remote prognosis for women at risk of renal and cardiovascular complications.

In this cohort study, there are several limitations. The blood pressure measurements beyond 6 weeks postpartum were only available for about half of the patients as many did not return for review beyond the six weeks. Data is not available on blood pressure at 12 weeks postpartum. Secondly there limited data on HELLP syndrome as liver enzymes were not assessed routinely for all patients either at initiation of the cohort or during subsequent follow-up. Resolution of hypertension in pregnancy may occur beyond 6 weeks postpartum. Therefore, assessment at 6 weeks (or before 12 weeks postpartum) may over-estimate the incidence of persistent hypertension. We acknowledge this limitation as a possible explanation for the high incidence of persistent hypertension in this cohort. The incidence of persistent hypertension is rather high compared to that of Sibai *et al*. [2]. This difference could be attributable to the short period of 6 weeks of follow-up for our participants compared to Sibai's study of 7 years. While endothelial damage is the most likely factor in the pathogenesis of pre-eclampsia, many factors associated with pre-eclampsia as well as their mechanisms are not well understood.

Women who develop chronic hypertension following pre-eclampsia/eclampsia are often unaware of their problem and future risk. In a study to determine the blood pressure and renal function seven years after pre-eclampsia/eclampsia complicated pregnancy [6], only 10 out of 28 women with chronic hypertension knew they were hypertensive. Microalbuminuria in such women is common, is closely related to residual renal dysfunction and is an early marker of glomerular disease [6,7]. Women who develop recurrent second trimester severe pre-eclampsia or eclampsia are more likely to have an underlying renal disease and tend to have a high risk of developing chronic hypertension on follow up. In a study to establish the likelihood of an underlying renal disease or chronic hypertension among women with a history of pre-eclampsia, 10% developed chronic hypertension and 2% had an underlying renal disease [8]. This highlights the importance of follow up for all women managed for pre- eclampsia/eclampsia who have high serum uric acid and creatinine levels after puerperium. This enables identification of women with underlying renal disease or ongoing renal damage, both of which impact negatively on subsequent health and pregnancy outcome.

The finding that serum creatinine was a predictor for persistent hypertension implies that we could use baseline serum creatinine levels when the patients are admitted to predict women who are at risk of developing chronic hypertension in future. Whereas some studies doubt the prognostic value of uric acid for maternal and fetal outcome in hypertensive pregnancy [9,10], hyperuricemia was associated with shorter gestations, lower birth weight, increased risk of preterm birth and risk of small-for-gestational-age infants, whether proteinuria was present or not [11]. This suggests that uric acid may be an important prognostic factor as effective as proteinuria at identifying gestational hypertensive pregnancies at increased risk [11].

Elevated uric acid levels in pre-eclamptic women may not simply be a marker of disease severity but possibly contribute directly to the pathogenesis of the disorder through ability of uric acid to promote inflammation, oxidative stress and endothelial dysfunction [12]. Increased adenosine may be a source of this uric acid [13]. Its levels are an indicator of ongoing oxidative stress [14] and could be responsible for the vascular injury of pre-eclampsia [15]. Though often considered an antioxidant, biochemical and in-vitro data indicate that non-crystalline, soluble uric acid can react to form radicals, increase lipid oxidation and induce various pro-oxidant effects in vascular cells [16]. From in-vitro and in- vivo studies, uric acid may contribute to endothelial dysfunction through inducing anti-proliferative effects on endothelium and impairing nitric oxide production in vascular smooth muscle cells (VSMCs) [16]. Hypertension develops through increased chemokine and cytokine expression, induction of the renin-angiotensin system and increased vascular C-reactive protein (CRP) expression [16]. This injury recovers within one month postpartum [17]. Improvement in glomerular filtration capacity is accompanied by recovery of hypertension to near-normal levels and significant improvement in albuminuria.

## Conclusion

Nearly one of every four mothers with pre-eclampsia/eclampsia are at risk of persistent hypertension at the end of the puerperium. Maternal age, serum creatinine and serum uric acid levels are predictors of persistent hypertension after the puerperium in women with pre-eclampsia and eclampsia.

## Competing interests

The authors declare that they have no competing interests.

## Authors' contributions

EBN was the graduate student who with DKK and MN as supervisors conceptualized and designed the study; carried out the data collection and follow up of the study participants, the data analysis and revised the draft manuscripts. MN contributed to design of the study, data analysis and interpretation and revision of the manuscripts. DKK was involved in the conception and design of the study, in the data analysis and interpretation, drafting of the initial manuscript and subsequent revision of the manuscripts. All co-authors approved the final manuscript.

## Pre-publication history

The pre-publication history for this paper can be accessed here:

http://www.biomedcentral.com/1471-2393/10/12/prepub
